# Neutrophil extracellular traps in upper respiratory tract secretions: insights into infectious and allergic rhinitis

**DOI:** 10.3389/fimmu.2023.1295921

**Published:** 2023-11-22

**Authors:** Marcin Zawrotniak, Magdalena Juszczak, Justyna Mosio-Wójcik, Maria Rapala-Kozik

**Affiliations:** ^1^ Department of Comparative Biochemistry and Bioanalytics, Faculty of Biochemistry, Biophysics and Biotechnology, Jagiellonian University, Krakow, Poland; ^2^ Doctoral School of Exact and Natural Sciences, Jagiellonian University, Krakow, Poland; ^3^ Independent Researcher, Krakow, Poland

**Keywords:** neutrophils, rhinitis, nasal secretion, NETs, elastase, myeloperoxidase

## Abstract

**Introduction:**

Neutrophil extracellular traps (NETs) are structures released by neutrophils in response to various infections. NETs have a biocidal role and have been demonstrated to be effective against bacteria, fungi, viruses, and parasites. Depending on the situation, NETs can protect the host from pathogen invasion or contribute to the development of autoimmune diseases such as cystic fibrosis and rheumatoid arthritis. In this study, we aimed to investigate the occurrence of NET as one of the components in upper respiratory tract secretions in infectious and allergic diseases.

**Methods:**

Nasal mucus was collected from donors diagnosed with infectious rhinitis or allergic rhinitis. The extracellular DNA content was determined using SytoxGreen staining, and the total protein pool was determined using the microBCA method. Micrococcal nuclease was used to digest the samples and ELISA was employed to identify the NET proteins. The enzymatic activity of elastase was determined.

**Results:**

Our findings showed that nasal mucus collected from patients with infectious rhinosinusitis contained extracellular DNA that could come from a variety of sources, responsible for increasing the density and viscosity of secretions, as well as NETs proteins. The identified enzymatic activity of NET elastase indicates the possible irritation of nasal tissues. However, the DNA content was not identified in the samples from allergic patients. In addition, we have shown in preliminary studies that therapy using N-acetylcysteine can liquefy nasal secretions.

**Discussion:**

The study suggests that the composition of nasal mucus varies according to the cause of mucosal irritation. The presence of DNA and NET proteins can have severe consequences for the therapeutic process prolonging treatment. The low viscosity of nasal mucus in allergic patients facilitates mucosal flushing and the removal of allergens. Understanding the occurrence and role of NETs in various respiratory diseases is critical for developing effective treatment strategies that consider the complex interaction between the immune system and pathogens. The results of this study suggest that NETs may be present in upper respiratory tract secretions with an infectious background, supporting basic defense mechanisms using eosinophils and EETs. Further research is needed to explore the potential of NETs as a therapeutic target in respiratory diseases.

## 1 Introduction

The upper respiratory tract is a primary barrier that separates the internal environment of the body from the air, which carries environmental factors such as dust, particles, microorganisms, and viruses (1). This system includes the nose and paranasal sinuses formed by epithelial cells, ciliated cells, and secretory glands that produce mucus. Such an anatomical configuration serves multiple key defensive and auxiliary functions during each inhalation. Primarily, its multilayered structure acts as a mechanical barrier preventing the uncontrolled spread and penetration of inhaled pollutants into deeper tissues (2). Mucus plays a crucial role in moisturizing and warming inhaled air, as well as immobilizing and removing particles and microorganisms. The composition of mucus varies depending on environmental conditions and health. However, it is predominantly comprised of water (95%) glycoproteins (2% – 3%), proteoglycans (0.1% – 0.5%), lipids (0.3% – 0.5%), proteins, and DNA (3, 4). The accumulation of foreign particles on the mucosal surface of the respiratory tract can irritate it, and microorganisms or viruses (e.g., the influenza virus) can lead to infections, resulting in inflammatory responses in the mucosa. Increased mucus production is a fundamental sign of this inflammatory state, which can lead to upper respiratory tract infections (5). Symptoms may also include blockages, sneezing, and itching, resulting from inflammation or nasal lining dysfunction. It is essential to note that there are at least two primary subtypes of nasal congestion: allergic rhinitis (AR) and infectious, each exhibiting different characteristics and mucus profiles (6). These alterations are often catalyzed by pro-inflammatory agents such as cytokines, chemokines, and other inflammatory mediators, which modulate epithelial cell activity and affect their secretory functions (2). Identifying the cause of inflammation is challenging, especially since it can be multifaceted. However, probable causes can be deduced from factors such as the severity of inflammation, symptoms, and tendencies (seasonal/recurrent or sporadic/constant), likely triggering factors (allergens, pathogens, etc.), and treatment efficacy. Moreover, the physicochemical properties of mucus, such as viscosity, density, and color, could hint at the underlying cause of inflammation (7).

The involvement of immune cells largely depends on the cause of the respiratory tract inflammation. Migration of these cells to the respiratory mucosa is facilitated due to its rich vascularity. The main fraction of cells involved in the response in the upper respiratory tract are eosinophils. Other immune cells involved in the nasal response are macrophages, mast cells, neutrophils, T and B lymphocytes, and dendritic cells.

Research on allergic rhinitis reveals that the basis of its symptoms is an IgE-mediated type I allergic reaction. In individuals with atopy, the contact of the mucous membrane with an allergen leads to the binding of the allergen with Immunoglobulin E (IgE) present on the surface of mast cells in the nasal mucosa or basophils in the peripheral blood. As a result of this phenomenon, histamine is released, along with other inflammatory mediators. This process results in the early phase of the allergic reaction, characterized by the appearance of symptoms such as watery rhinorrhea, sneezing, and itching. Later, nasal congestion occurs as a consequence of this reaction. Additionally, released cytokines and chemotactic factors, like IL-4, IL-5, and IL-13 contribute to the accumulation of various cells, including eosinophils, mast cells, and lymphocytes in the nasal mucosa. This stage is known as the late phase of the allergic reaction, which manifests itself 6-12 hours after exposure to the allergen. It is also worth emphasizing that allergic rhinitis is divided into two categories depending on the type of allergen and the timing of the symptoms. It can occur seasonally or throughout the year, depending on the type of allergen responsible for triggering the allergic reaction (1, 2, 5).

A different process is observed in infectious-originating inflammation. The most common cause of acute rhinitis is viral infections caused by coronaviruses, adenoviruses, influenza viruses, and other respiratory system virus types (8, 9). However, bacterial and fungal infections are also observed, causing thick mucus. Increased infiltration of phagocytic cells, such as neutrophils and macrophages, is seen in both nasal secretions and tissues, primarily in response to the production of pro-inflammatory cytokines like IL-1β, TNF-α, IL-4, IL-5, IL-13, and IFN-γ (in the case of viral infections) (10). Furthermore, the presence of polyps induces eosinophil recruitment to the inflamed mucosa, leading to an increase in mucus viscosity and density (6).

The role of neutrophils in nasal inflammation is still poorly understood. Nasal swabs and tissue biopsy analyses have shown the presence of neutrophils, but their direct function in the upper respiratory tract remains unconfirmed (2, 11). It is conceivable that neutrophils play a dual role in mediating the phagocytosis of pathogens and immunogenic particulates, as well as modulating the inflammatory response. These functions are achieved through granular antimicrobial proteins and response mechanisms to infections, which involve pathogen removal via phagocytosis, immobilization, and combat using neutrophil extracellular traps (NETs) (12). NETs represent one of the effective extracellular mechanisms that restrict microbial spread from infection sites. This is feasible due to the structure of these entities, comprising DNA strands released from the cell nucleus adorned with numerous granular proteins, such as elastase, myeloperoxidase, and others. Another source of extracellular DNA may be the mitochondrial DNA (mtDNA) (13). The mtDNA release mechanism, unlike the classical pathway, is not associated with direct cell death. The electrostatic charge of DNA effectively immobilizes pathogens, whereas the enzymes cause structural damage to the invading cells (14, 15). However, NETs can also have adverse effects on host tissues and body functionality. For instance, NETs play a role in clot formation, leading to thrombosis (16). They also have a significant role in lower respiratory tract diseases. In cystic fibrosis (CF), the presence of neutrophil DNA in secretions, which is responsible for increasing their density and viscosity, has been observed, thus impeding their removal (17). Therefore, DNase inhalation is used as a therapeutic method to liquefy accumulated secretion (18). Daily administration of nebulized recombinant human deoxyribonuclease (rhDNase), is extensively utilized as a primary mucolytic treatment for individuals suffering from CF. Research has demonstrated its effectiveness in diminishing the thickness of CF patients’ sputum, consequently facilitating the expulsion of secretions. Clinical trials have indicated that rhDNase is a well-received therapeutic intervention, leading to an enhancement in pulmonary performance and a reduction in respiratory exacerbations (19). Moreover, proteolytic enzymes and myeloperoxidase present in NETs contribute to epithelial damage and further inflammatory progression, leading to acute lung injury (ALI) (20). A study by Hwang et al. pointed to the potential presence of neutrophil eDNA in chronic rhinosinusitis (CRS) due to epithelial damage (2). Ueki et al. indicated that eosinophils also release extracellular traps (EETs) in the upper respiratory space, which might enhance mucus viscosity (21). The main protein components of EETs are major basic protein (MBP), eosinophil peroxidase (EPX) and eosinophil cationic protein (ECP).

Currently, there are no definitive treatments for upper respiratory inflammation. The proposed therapies (excluding targeted antibiotic therapy) focus on symptomatic relief and alleviation of accompanying discomforts. Additionally, to improve patient comfort, the mucolytic drug N-acetylcysteine (NAC) is used to liquefy the secretion, though its impact on inflammation progression appears negligible (22). However, NAC might modulate the neutrophil and eosinophil response in the form of netosis (23) and EETosis (24). In addition, NAC has been shown to be effective in the treatment of ALI/ARDS in the course of COVID-19, helping to control inflammation, oxidation levels and improve patient health (25).

The role of neutrophils in upper respiratory tract infection is poorly understood, and the emergence of NETs has not yet been described. This paper describes the unknown role of NETs in upper respiratory tract secretions, identifying one mechanism that reduces patients’ quality of life and thus a potential therapeutic target. In our study, we focused on comparing the content of NETs in the upper airway secretions of patients suffering from infectious and allergic airway obstruction. We determined the role of NETs in mucus thickening and assessed the content of protein components and proteolytic activity of elastase and MPO in the samples. Furthermore, we identified the effect of NAC-mediated therapy on the release of NETs in the upper airway space.

## 2 Materials and methods

### 2.1 Sample collection

Secretion samples from the upper respiratory tract were obtained as waste material following routine diagnostic tests for the presence of pathogenic microorganisms in the upper respiratory tract secretions of patients. These patients were diagnosed with upper respiratory tract inflammation of either infectious or allergic origin based on a medical examination, following current guidelines, the season of upper respiratory infection or allergies, and the determination of IgE antibody titers. Patients were identified by screening for bacterial and viral infection (influenza virus A and B, as well as selected coronavirus variants). Negative samples, confirmed by determination of IgE levels, were classified as allergic. The infectious group consisted of 32 samples, while the allergic group comprised 30 samples. Due to the anonymization of the samples, additional individual information such as age, gender, onset time of disease symptoms, and other parameters were not included. The samples were obtained from both male and female individuals aged 18-52 years who were not taking anti-inflammatory, immunosuppressive, or any other drugs that could affect the effectiveness of the immune system. Samples from infectious patients were characterized by high viscosity (HVS), whereas those from allergic patients had low viscosity (LVS).

For laboratory studies, samples were collected by aspiration using a sterile pipette (Sarsted, Nümbrecht, Germany) and then transferred to a disposable plastic tube (BD, Franklin Lakes, NJ, USA). In the case of high-viscosity samples, the secretion in the respiratory tract was liquefied by introducing a small volume of saline solution (Polpharma, Warsaw, Poland). Containers were stored at a temperature of -20°C for a maximum period of 2 months.

### 2.2 Staining and microscopy analyses of samples

For staining of samples for microscopic imaging, 50 µl of the sample fixed with 3.8% paraformaldehyde (Sigma-Aldrich, Waltham, MA, USA) was transferred onto a microscope slide, then heated to evaporate the solution (70°C, 10 min). For the degradation of nucleic acids, 10 µl of DNase I solution (Roche, Basel, Switzerland) at a concentration of 100 U/ml in PBS supplemented with MgCl_2_ (Biowest, Nuaillé, France) was added to the slide before evaporation, and incubated for 30 minutes at 37°C. Fixed samples were labeled with primary anti-elastase antibody (1:200) (Abcam, Cambridge, UK), anti-myeloperoxidase antibody (1:400) (Abcam) or anti-Eosinophil Major Basic Protein antibody (1:400) (Sigma-aldrich) at 4°C overnight, washed three times with PBS, and then incubated with secondary Alexa Fluor 555‐conjugated antibody (Abcam) for 1h at RT. Controls were samples labeled only with secondary antibodies. The samples were washed and co-stained with 1µM SytoxGreen (Sigma-Aldrich) for 2 min, and then covered with a cover slip using Fluorescent Mounting Media (Sigma-Aldrich). Such prepared samples were microscopically imaged using Olympus IX73 (Evident, Tokyo, Japan).

### 2.3 Analysis of extracellular DNA concentration in samples

400 μl of each sample was transferred to new tubes. A 100 μl aliquot of micrococcal nuclease (MNase) at a concentration of 1 U/ml (Roche) was added to each tube and incubated at 37°C for 20 min to digest extracellular DNA. The nuclease activity was stopped by the addition of 5 mM EDTA. The tubes were centrifuged for 5 min at 300 × g and the supernatants were collected. SytoxGreen was added to supernatants at a final concentration of 1 μM and 50 μl of each sample was transferred into a 96-well microplate to measure fluorescence (EX/EM = 495/525 nm) using a Synergy H1 microplate reader (Biotek, Winooski, VT, USA).

### 2.4 Analysis of the total protein concentration

#### 2.4.1 Micro-BCA protein assay

Protein concentrations in the samples were determined using the micro-BCA protein assay kit (Sigma-Aldrich), following the manufacturer’s protocol. In brief, 150 μl of samples (treated with DNase I as described above) or protein standards (BSA) were pipetted into a 96-well microplate. Following the addition of the working reagent, the plate was incubated at 37°C for 2 hours. After incubation, the absorbance of each well was recorded. Protein concentrations of the samples were interpolated from a standard curve generated from known protein standards provided with the kit.

#### 2.4.2 Bradford protein assay

For measurements of protein concentration over time, the Bradford assay was used (26). Briefly, a 4 μl volume of each sample was mixed with 200 μl of Bradford reagent in a 96-well microplate. After 5 min of incubation at RT, the absorbance at 595 nm was measured using a Synergy H1 microplate reader (Biotek). Standard curves were constructed using known concentrations of bovine serum albumin (BSA).

### 2.5 Determination of elastase and myeloperoxidase concentrations by ELISA method

The quantitative determination of HNE or MPO was performed using a Human ELANE or MPO ELISA Kit (Wuhan Fine Biotech Co., Ltd., Wuhan, China). The samples were treated with DNase as described above. The supernatant was transferred into anti-HNE or anti-MPO pre-coated wells of plate, and the manufacturer’s instruction was followed.

### 2.6 Elastase activity analysis

The activity of elastase was measured in selected samples that had similar protease concentrations. Some of the samples were subjected to degradation with DNase I, as previously described. Samples were analyzed following the manufacturer’s protocol (Elastase Substrate I, Sigma-Aldrich). In brief, 2 μl of the samples were transferred to 48 μl of buffer, followed by the addition of 50 μl of substrate and incubation at 37°C, measuring fluorescence at Ex/Em = 380/500 nm every 2 minutes. The obtained results were presented as a percentage of the positive control.

### 2.7 Statistical analysis

Statistical analysis was performed with the GraphPad Prism 8 (GraphPad Software, CA, U.S.A.). The statistical significance was assessed by t-Student or one-way ANOVA followed by a Tukey’s test for multiple comparisons.

## 3 Results

### 3.1 Upper respiratory tract secretions contain significant amounts of nucleic acids

Research on the presence of NETs in the secretions of the upper respiratory tract was conducted on samples obtained from voluntary donors. During the heightened season of viral-based upper respiratory tract infections, a medical examination confirmed an upper respiratory tract infection in these donors. Samples were also obtained from donors suffering from seasonal allergies triggered by plant pollen, confirmed by the diagnostic presence of IgE class antibodies.

The obtained samples significantly varied in density between the two groups. Samples from infectious donors were characterized by high viscosity (high viscosity secretions, HVS) and showed stretching typical for nucleic acids. In contrast, samples from allergic donors behaved as aqueous solutions, showing no increased adhesion to plastic surfaces, and were characterized by low viscosity (low viscosity secretions, LVS). Due to the similarity of the HVS samples to solutions of isolated DNA, the secretions from both research groups were microscopically analyzed for the content of extracellular nucleic acids. For this purpose, the samples were transferred to wells of a 96-well plate, then stained using a DNA marker (SytoxGreen) and imaged microscopically.

**Figure 1:**
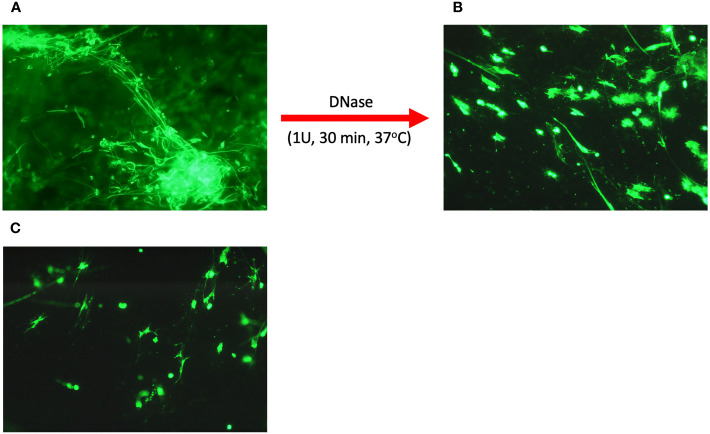
Microscopic analysis of eDNA in upper respiratory tract secretions. Secretion samples from patients suffering from infectious upper respiratory tract inflammation **(A)** and allergic inflammation **(B)** were fixed on a basic slide and then stained with eDNA using SytoxGreen. In addition, a secretion sample from an infectious donor was treated with 1U DNase I for 30 minutes **(C)**. Samples were visualized microscopically using an Olympus IX73 microscope.

Microscopic imaging revealed that in the group of samples from infectious donors, there was a large amount of extracellular nucleic acids, whose structure resembled stretched NETs (**Figure 1A**). Analysis of LVS samples from allergic donors showed only small amounts of extracellular nucleic acids, not forming characteristic structures, but rather dense structures around cell remnants (**Figure 1B**).

It seems that the nucleic acids present in the HVS samples are responsible for the dense structure of the secretion during infection. To confirm their role, the samples were treated with 1U of DNase for 30 minutes at 37°C, and then microscopically imaged. A significant decrease in the amount of extracellular DNA was observed, along with the degradation of long NET-like structures (**Figure 1C**). In addition, nucleic acid degradation led to the loosening of the dense and viscous structure of secretion, liquefying it (**Figures 1A, B**, epp tube). This proves that the presence of nucleic acids is a crucial factor determining the physicochemical properties of the secretion, and thus indirectly determines its functions related to the immobilization of allergens and pathogens, similar to the role of NETs networks in tissues or the lower respiratory tract.

**Figure 2:**
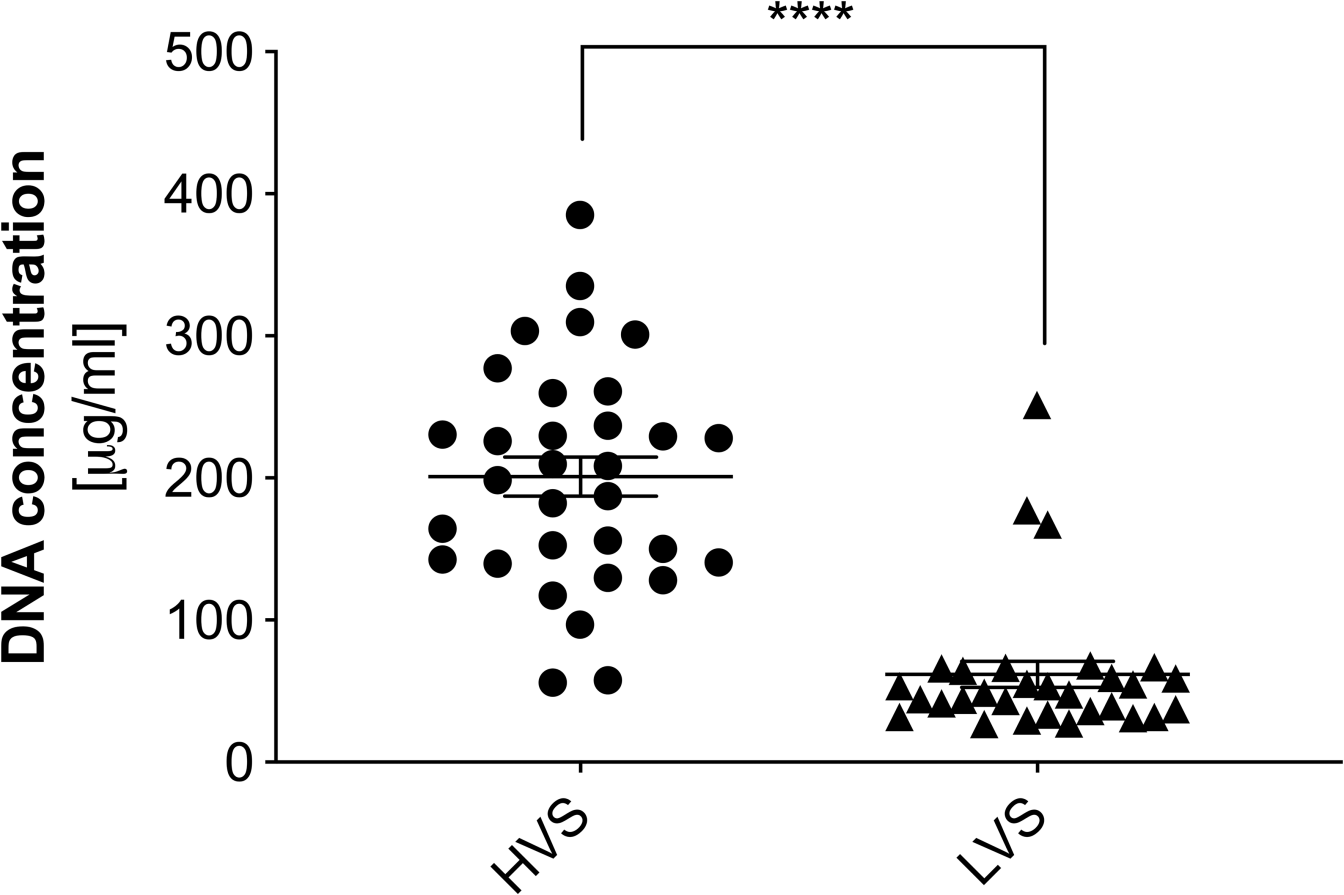
The concentration of extracellular DNA in high and low-viscosity samples. The concentration of DNA in samples from the HVS and LVS donor groups was quantified using a fluorescence method. Samples were degraded into short DNA fragments using micrococcal nuclease (MNase) and then stained with SytoxGreen. Fluorescence intensity was measured using a Biotek Synergy H1 microplate reader. The results presented a mean ± SEM of all samples in duplicate. Asterisks indicate statistically significant differences between samples (****p < 0.001).

For a quantitative evaluation of the nucleic acid content in the studied samples, suspensions were digested using micrococcal nuclease (MNase). The use of MNase allowed for the liquefaction of HVS samples by partial degradation of the DNA strands and the obtaining of short nucleic acid fragments, while retaining the ability to bind fluorescent nucleic acid dyes. As a result, the obtained preparations constituted homogeneous solutions enabling further analysis. In the next step, the samples were stained with the fluorescent dye PicoGreen, and the concentration of dsDNA was determined using a calibration curve based on λ-DNA. The obtained results are presented in **Figure 2**. A high nucleic acid content was confirmed in the HVS samples, averaging 200 µg/ml of the sample, but the concentration varied significantly. In the LVS samples, the DNA concentration was four times lower (average 50 µg/ml), with a simultaneously higher homogeneity of results. Three LVS samples showed a significantly higher DNA concentration, comparable to the content observed in the HVS group. This might be associated with an undiagnosed infection or a mixed type of inflammation and incorrect assignment of donors to specific groups. However, due to the number of samples analyzed, they do not significantly affect the obtained results. The result confirms microscopic observations that the secretion of the upper respiratory tract during CRS contains a significantly higher concentration of extracellular nucleic acids than in the LVS samples.

### 3.2 Proteins present in secretion are associated with nucleic acids

**Figure 3:**
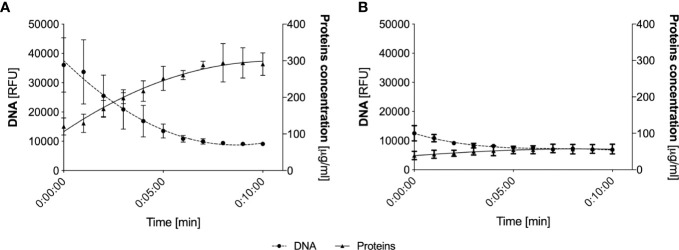
Analysis of protein release from eDNA following nucleic acid degradation. Representative HVS **(A)** and LVS **(B)** samples were degraded using DNAase I, analyzing DNA levels at 1-minute intervals (fluorimetric assays, SytoxGreen) and total protein concentration (Bradford method). The data represent the average value from the analysis of three randomly selected samples.

As part of the further analysis of the composition of upper respiratory tract secretions, we focused on determining the total protein concentration in HVS and LVS samples using the micro-BCA method. In NETs, a significant number of proteins are associated with extracellular DNA, on the one hand, limiting its spread, and on the other, inhibiting its enzymatic activity (27, 28). To release proteins from the DNA and thus obtain a homogeneous solution, the samples were treated with DNase. The method of DNA degradation before the protein analysis of the samples has been previously described (29). During degradation, at 1-minute intervals, the DNA level was determined by the fluorometric method and samples were also taken to determine the protein concentration in the solution using a Bradford method. Such analyses allowed for the comparison of protein interactions with extracellular DNA in samples from two donor groups and confirmed the obtaining of a stable final concentration of released proteins (**Figure 3**). As suspected, the degradation of nucleic acids in DNA-rich samples (HVS) resulted in the release of proteins associated with nucleic acids within 5 minutes. As a result of degradation, the total concentration of proteins increased from 150 μg/ml to about 280 μg/ml, resulting in an almost twofold change in concentration. The increase in protein concentration was correlated with a simultaneous decrease in DNA amount, reaching a plateau after 5 minutes. These results indicate that proteins in HVS samples are associated with extracellular DNA, suggesting that the structures observed microscopically may represent ETs released by neutrophils or other immune cells, like macrophages, DC-cells, or eosinophils. The same analysis conducted for LVS samples showed only an insignificant change in protein and DNA concentration.

**Figure 4:**
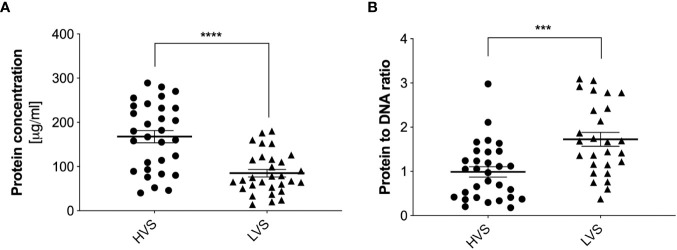
Quantitative determination of total protein concentration in HVS and LVS samples. The donor samples of the two groups were treated with DNase I for 10 minutes, and then the total protein concentration was determined using the micro-BCA method **(A)**. Absorbance was measured using a Biotek Synergy H1 microplate reader. The ratio of total protein to DNA concentration is shown in **(B)**. The results presented a mean ± SEM of all samples in duplicate. Asterisks indicate statistically significant differences between samples (***p < 0.005, ****p < 0.001).

These analyses were performed for three randomly selected samples from each group. To analyze the secretions collected from all donors, the samples were treated with 1U of DNase I for 10 minutes and then protein concentrations were determined similarly using the micro-BCA method. For HVS samples, a large variation in protein concentration was observed, with an average value of about 180 µg/ml (**Figure 4**). In contrast, LVS samples showed a more consistent profile, with an average protein concentration of about 70 µg/ml. The high variability in protein concentration among individual donors of the HVS group correlates with the variability in DNA concentration, which may be related to different stages of infection, its duration, and varying responses to the ongoing infection. The difference in protein concentration between HVS and LVS samples suggests that infectious processes may lead to increased production or release of proteins in the upper respiratory tract compared to allergic reactions. The protein-to-DNA ratio in HVS samples is similar to the value characteristic of NETs (29), which may suggest similarities in their formation mechanisms or composition. However, it should be considered that the calculated ratio of DNA to protein concentrations applies not only to NETs components, but also to nucleic acids and proteins from different sources present in the secretion, which makes it impossible to precisely determine the ratio and compare the results with NETs obtained *in vitro*. Considering that the total pool of proteins in the obtained samples was analyzed, the increased concentration in HVS samples may indicate an intense immune response to infection, and protein release also by epithelial or pathogens cells at the site of infection.

The identification of specific neutrophil proteins, such as human neutrophil elastase (HNE) and myeloperoxidase (MPO), allows for the confirmation that neutrophils have released granular proteins through netosis or degranulation at the site of inflammation. To rule out degranulation as the source of extracellular proteins, microscopic imaging of preparations with stained neutrophil proteins and DNA is commonly used. The presence of the analyzed proteins associated with DNA strands is sufficient to confirm the presence of NETs in the preparations.

### 3.3 NETs present in upper respiratory tract secretions increase their viscosity

**Figure 5:**
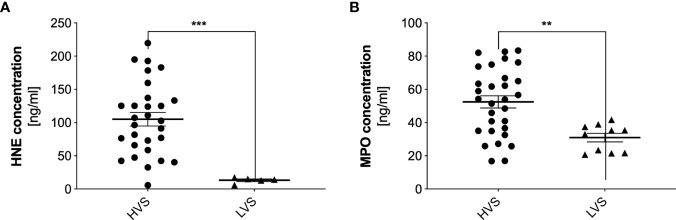
Concentrations of human neutrophil elastase and myeloperoxidase in donor samples. HVS and LVS group secretion samples were treated with DNase I for 10 minutes, and then HNE **(A)** and MPO **(B)** concentrations were determined by ELISA. Absorbance was measured using a Biotek Synergy H1 microplate reader. The results presented a mean ± SEM of all samples in duplicate. The number of samples used for analysis was: N_HVS_= 32, N_LVS_=30; due to low concentrations of the proteins in the analyzed samples, below the method’s detection limit, the results for HNE show N_HVS_= 29, N_LVS_=5, but for MPO - N_HVS_= 30, N_LVS_=10. Asterisks indicate statistically significant differences between samples and negative control (**p < 0.01; ***p < 0.005).

For further research, HNE and MPO were selected as a NET-specific protein, due to their high content in NETs (29). The concentration of these proteins in samples from both donor groups was analyzed by ELISA, where samples previously treated with DNase were used (30). HNE concentration in the HVS sample group was highly varied, ranging from undetectable to 230 ng/ml (**Figure 5A**). The average concentration for the studied group is 105 ng/ml. Determining the concentration of elastase for LVS was possible for only five samples and averaged 13 ng/ml. For the remaining samples, the concentration was below the detection capability of the method. The significant difference in mean concentration between the two analyzed groups suggested that only in the HVS group was HNE released into the extracellular space, which was due to differential recruitment or activation of neutrophils by pro-inflammatory factors. The MPO concentration in the two groups also varied, but in the HVS sample group, the MPO concentration was 52 ng/ml, while in the LVS samples, it was 31 ng/ml, so the difference was not as significant as in the HNE (**Figure 5B**). Similarly, the results for the HVS group showed great variability, in contrast to the LVS samples. The presence of MPO in the allergic donor group may be due to the varying source of this enzyme released into the extracellular space.

**Figure 6:**
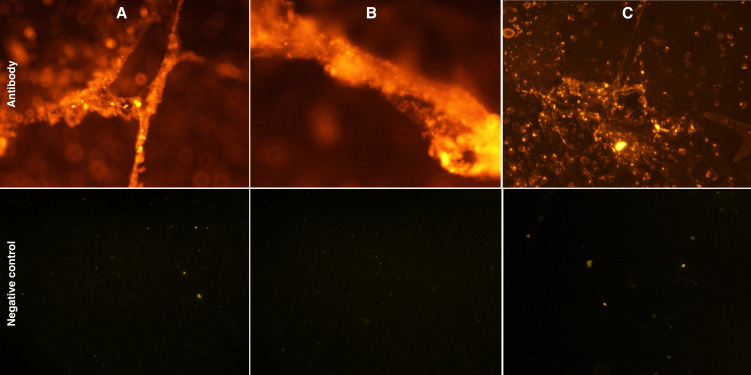
Microscopic analysis of NETs- and EETs associated human neutrophil elastase,myeloperoxidase and human eosinophil major basic protein (MBP). Samples of HVS secretion fixed with 4% PFA on a primary slide were incubated with anti-HNE **(A)**,anti-MPO **(B)** and anti-MBP **(C)** antibodies. The slides were then labeled with Alexa Fluor 555-conjugated secondary antibodies and imaged microscopically using an Olympus IX73 microscope. **(A–C)** are representative of one of 10 samples evaluated.

To confirm that HNE and MPO are released into the extracellular space as a result of netosis, selected HVS preparations were labeled with fluorescent antibodies and microscopically imaged. The obtained results demonstrate that both HNE (**Figure 6A**) and MPO (**Figure 6B**) are associated with extracellular DNA fibers, thus confirming the presence of NETs in the secretions of the upper respiratory tract during infectious-origin CRS. In addition, MBP protein, a component of the network of EETs released by eosinophils, was also labeled. The result showed (**Figure 6C**) that in the composition of upper respiratory tract secretions, EETs are present simultaneously with NETs, as previously demonstrated (21).

**Figure 7:**
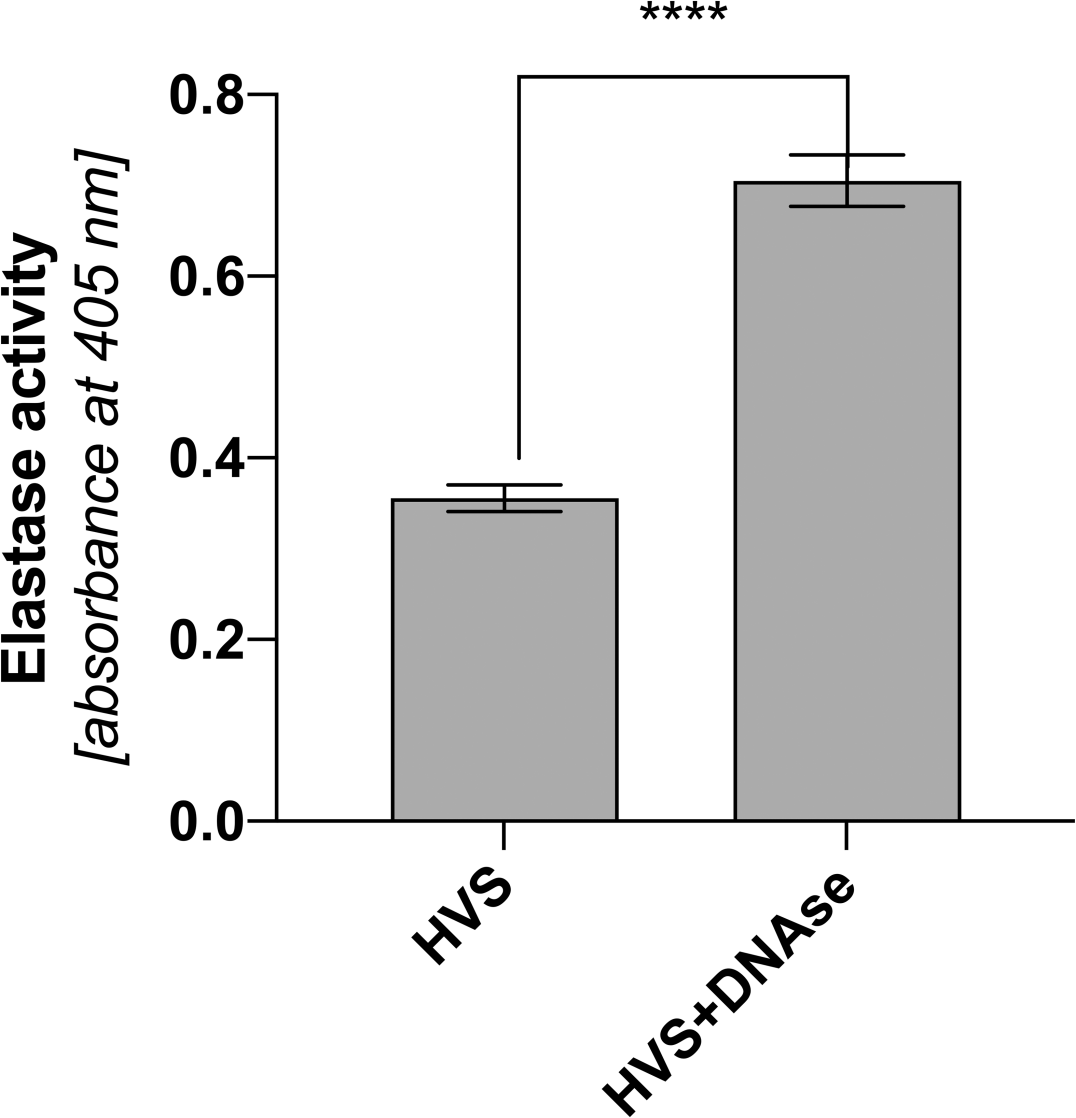
Proteolytic activity of elastase in upper respiratory tract secretions of infectious patients. Analysis of the proteolytic activity of elastase was performed on HVS secretion samples and HVS samples subjected to DNA degradation. The measurement was based on the absorbance method of analyzing the resulting product using a Biotek Synergy H1 microplate reader. The results presented a mean ± SEM of selected samples analyzed in duplicate. Asterisks indicate statistically significant differences between samples (****p < 0.001).

Complexation of HNE and DNA leads to inhibition of protease activity (27, 31). On the one hand, this action limits the biocidal properties of elastase, and, on the other, it reduces the proteolytic damage to the host’s tissues. The nasal mucous membrane is particularly sensitive to prolonged inflammation, which is why in the next stage of research, the proteolytic activity of elastase was determined in HVS representative samples. To compare the effect of DNA interaction on elastase activity, its activity was determined in each of the samples with or without prior degradation by DNase, as previously described. The activity was determined using a colorimetric method, comparing the activity between individual samples. The obtained results indicate that the elastase associated with NETs shows activity but is reduced (**Figure 7**). DNA degradation and protease release double its activity. The reduction in activity can potentially have positive effects on the host organism, limiting the proteolytically induced inflammatory state and thus further thickening the produced secretion.

### 3.4 NAC causes a reduction of the amount of NETs in upper respiratory tract secretions

**Figure 8:**
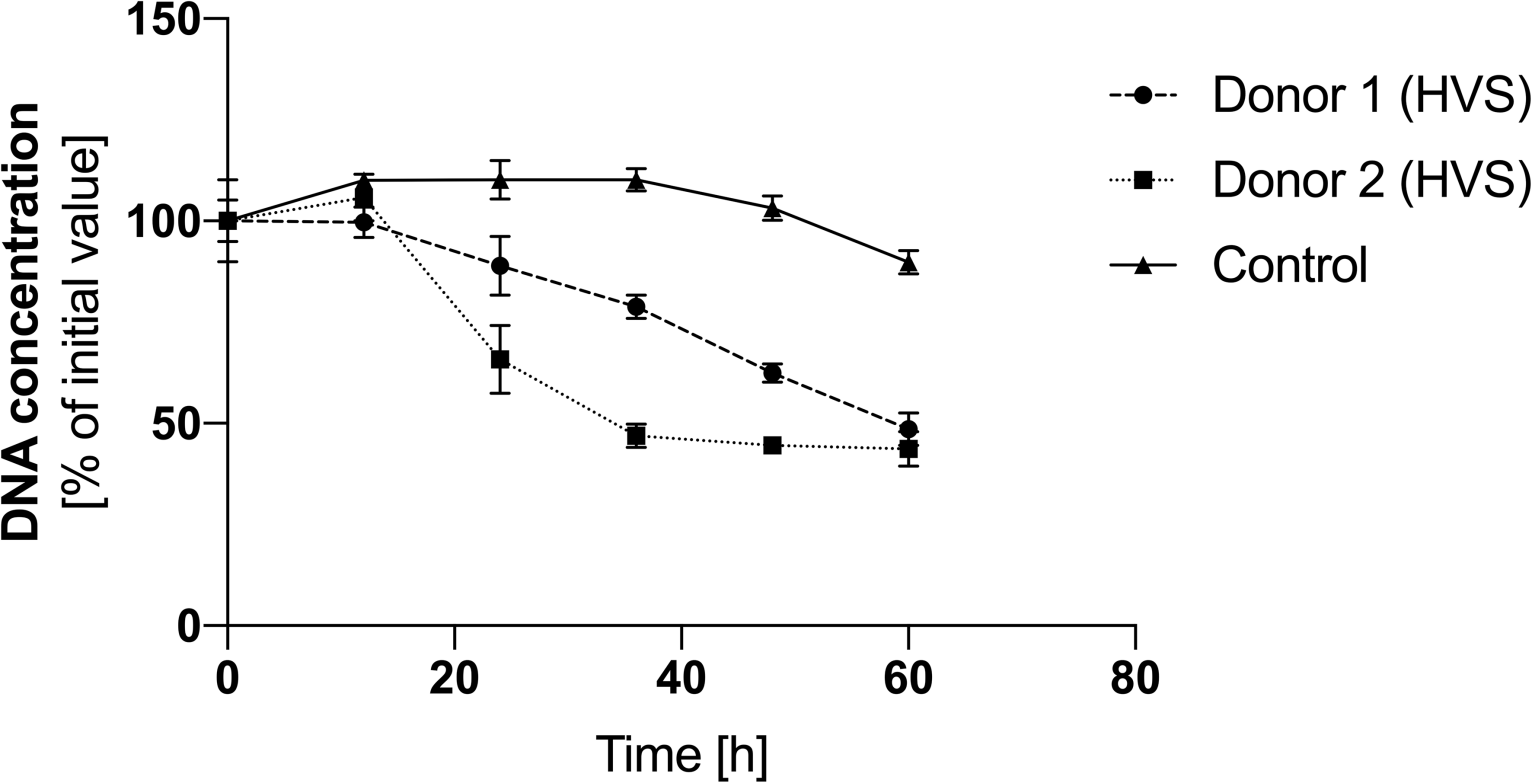
The effect of NAC on the level of NETs in HVS secretion. Samples of secretion were taken every 12 hours from patients starting therapy with N-acetylcysteine at a dose of 200 mg/12 h to analyze the level of eDNA. DNA was degraded into short fragments using MNase, and then the level of nucleic acids was quantitatively determined using the fluorimetric method with SytoxGreen. The results presented a mean ± SEM of selected samples in duplicate.

Our previously published studies showed that under *in vitro* conditions, N-acetylcysteine, by inhibiting ROS production mechanisms in neutrophils, inhibits the ROS-dependent netosis pathway (23). Two of all donors in the HVS group started oral administration of NAC at a dose of 200 mg every 12 hours as part of CRS therapy. To analyze the effect of NAC on the level of NETs in the secretion, it was collected for 60 hours, at 12-hour intervals, and then, using a methodology analogous to the one described above, the level of NETs in individual samples was determined. Time zero was the first drug administration. Due to the lack of donors taking NAC in the LVS group and the low DNA level, no analysis was conducted for this group. The control was an HVS group donor who did not take any drugs during the analogous period and was not undergoing treatment. The obtained results showed that NAC administration can significantly reduce the amount of NETs in the secretion of the upper respiratory tract (**Figure 8**). During the first 12 hours after taking the drug, the level of extracellular NETs did not change. After 24 hours, there was a decrease of about 30% in the level of NETs in the analyzed secretions in donors taking NAC. Every subsequent 12 hours, the DNA content in both donors taking NAC systematically decreased, reaching 40% of the initial concentration after 60 hours. The DNA level in the donor not taking NAC, and not undergoing pharmacological therapy for 36 hours, increased to 110% and then dropped to 90% of the initial value. The obtained results indicate that NAC reduces the level of NETs in the secretion of the upper respiratory tract during CRS. Due to the nature of the research and the significantly limited research group, which was not normalized, e.g. due to the duration of ailments, age, health condition, accompanying symptoms, etc., the presented results are of a pilot/preliminary nature.

## 4 Discussion

Upper airway obstruction can be caused by accumulated secretions that impede the free flow of air through the nose and ventilation of the paranasal sinuses. Increased secretion production is associated with overactivity of the epithelial and glandular cells that form the mucous membrane. There are many causes of this overactivity, ranging from allergic factors and pathogenic infections to idiopathic causes. However, all of them lead to the development of inflammation, swelling, and the release of pro-inflammatory cytokines that recruit immune cells to the mucosal area (1, 5, 32). In pathogen-induced CRS, phagocytic cells such as macrophages and neutrophils, as well as mast cells, play an important role (6). Secretions appearing in the respiratory tract are characterized by high viscosity, making their removal a challenge. Inflammation caused by allergic agents, such as pollen, causes primary migration of eosinophils and an increase in IgE levels, and the resulting secretion is usually fluid (1, 33).

In our work, we focused on identifying NETs and their selected components in two types of upper respiratory tract secretions. The study showed that secretions from patients with pathogen-induced development of inflammation are characterized by a high content of nucleic acids, forming structures characteristic of NETs on microscopic imaging, i.e., cloud-like structures and long DNA fibers (34). In contrast, no significant amounts of nucleic acids were observed in samples from allergic patients. The high variability in DNA concentration for the HVS group may be due to several factors, most likely individual donor variability, resulting in the differential potential of cells to release ETs. Additionally, a broad spectrum of stimulatory factors at the site of inflammation may activate cells and subsequent mechanisms of ETosis at different levels. The varying amounts of DNA in the secretion can modulate its viscosity and density.

The DNA degradation we performed showed that the removal of DNA strands leads to a loosening and liquefaction of the secretion structure. Thus, nucleic acids are largely responsible for its thickening and increased viscosity. Similarly, NETs, released in the lower respiratory tract during cystic fibrosis, probably play a similar role as an adverse side effect for the body, thickening secretions and making them more difficult to clear (35, 36). As part of the accepted symptomatic treatment, DNase is administered by inhalation to liquefy stagnant secretions. There may be many potential sources of eDNA in the upper respiratory tract, but neutrophils and other cells that release extracellular nucleic acid-based traps appear to be the main source. The presence of neutrophils has been confirmed in the sinus mucosa of patients suffering from CRS without developed polyps, as well as in smears of patients with inflammation of idiopathic origin (2). In addition, the presence of neutrophils and eosinophils has been demonstrated in the mucosa of patients with symptoms of non-allergic rhinitis (11, 21, 37–39). The presence of NET has also been confirmed in the secretions of patients suffering from acute CRS (2).

Proteomic analyses of secretions by various research groups indicate the presence of a wide spectrum of proteins of various origins, including lysozyme, myeloperoxidase, eosinophil cationic protein, interleukins, lactoferrin, defensins, elastase, etc. (3, 40–42). It is noteworthy that many proteins exhibit strong bactericidal activity, suggesting the defensive nature of secretions against the presence of potential pathogenic microorganisms. The origin of the proteins in the secretion has not been conclusively confirmed, but previous studies point to several immune cells, mainly neutrophils and eosinophils, as potential sources of these components. Our analyses of total protein concentrations showed that HVS samples contain, on average, twice as much total protein as LVS, partially bound to eDNA. However, this difference is not as large as the difference in DNA concentration between the two groups analyzed. The higher protein concentration in HVS samples may be due to both higher epithelial and immune cell activity, greater accumulation of these cells, and multiple sources of proteins and their retention in nucleic acids (2). During the allergic response, the mechanism of neutrophil degranulation and eosinophilia may be activated (43), which may explain the elevated protein levels without the presence of significant amounts of NETs. Although DNA and protein concentrations are higher in HVS, however, the protein to DNA ratio is higher for LVS samples. This is due to the low concentration of DNA in LVS samples. This result indicates that the low-viscosity secretion contains much fewer DNA from various sources, including NETs and EETs. The source of protein in LVS can be immune cells, mainly eosinophils that release proteins into the extracellular space, as well as epithelial cells or plasma proteins. Identification of protein sources in specific disease entities, although highly complex, can be valuable diagnostic information. The protein to DNA results presented in this paper may serve as one indication suggesting a cause (infection vs. allergy) of upper airway obstruction, but this requires further verification.

Microscopic identification of neutrophil elastase and MPO bound to eDNA confirmed that the observed structures are NETs released by neutrophils. Neutrophils can be the unique source of elastase, and this DNA-bound protease is present only in NETs after the protein has migrated from the granule to the cell nucleus (44). Quantitative analysis showed that elastase is present only in HVS samples, while MPO is also present in LVS samples. This result indicates that during an allergic inflammatory reaction, neither NET nor elastase is released by degranulation. However, the source of MPO may be other cells, such as eosinophils that release the enzyme by degranulation, the presence of which in allergic patients has been confirmed (43). In addition, MPO is a marker of cell activation in acute sinusitis (45). Urban et al. showed that *in vitro*, the ratio of total maximal protein to DNA concentration in NETs is about 1.6 (29). However, in the case of secretion analysis, both proteins and DNA may come from different sources, and the level of response may vary, so the ratio will not be maintained.

The presence of NETs in nasal secretions appears to be defensive and plays roles directly related to pathogen elimination. The eDNA immobilizes pathogens, limiting their spread into the lower respiratory tract (36). The role of elastase in NET is proteolytic degradation of pathogen proteins, while MPO is responsible for catalyzing the production of hypochlorous acid, which is highly toxic to microorganisms. Despite their undeniable advantages in terms of defense functions, these proteins can lead to damage to the host epithelium, resulting in chronic inflammation (16). The presence of NETs leads to the stimulation of epithelial cells and the release of pro-inflammatory chemokines, responsible for the further recruitment of phagocytes (2), resulting in increased inflammation (46). NETs are cytotoxic to airway epithelial and endothelial cells (47), leading to acute lung injury (48). Our results showed that elastase activity in secretions is inhibited by complexing with DNA. DNA degradation and elastase release significantly increase elastase activity, as shown in other studies (27, 30). Such inhibition by nucleic acids, however, may be beneficial to the host, reducing the negative effects of NET and regulating the course of inflammation.

The mechanism of netosis and NETs structures seems to be excellent targets for complementary therapy for CRS and other diseases leading to upper airway obstruction. Both controlling the mechanism of netosis and degrading already released NETs can help liquefy upper airway secretions, thereby improving patients’ quality of life. The use of DNase to liquefy lower airway secretions may be equally effective for the nose and sinuses, but this therapy does not address the mechanism that leads to the thickening of secretions. Our published findings indicate that N-acetylcysteine, by inhibiting ROS in neutrophils, can inhibit or limit NET release in the ROS-dependent netosis pathway (23). In our analysis, we demonstrated that patients on standard CRS therapy with NAC experienced a significant reduction in NETs in their secretion within a day. NAC was administered orally, so the drug’s effect on NETs was not direct, as with inhaled DNase. Due to the limitation of the study group to two donors and one control, the results obtained are indicative and require further verification, but suggest possible directions for further clinical research. Similar results were observed for eosinophils, where NAC through ROS inhibition blocked the release of EETs (24). Thus, the effect observed in our study may be the sum effect of NAC on neutrophils, as well as eosinophils, which are the main leukocyte fraction in the upper respiratory tract. Other studies on the effects of NAC on the course of airway inflammation have shown that NAC, on the one hand, acts as a reversible inhibitor of the beating frequency of human cilia *in vitro* (49), and, on the other hand, in combination with antibiotic therapy, significantly facilitates the removal of mucus from the airways and supports the effect of antibiotics (22).

The presence of NETs, as well as eDNA of other origin, including eosin EETs, represents an interesting diagnostic and therapeutic target. The results presented in this paper are an important prelude to further studies on the role of neutrophils and NETs in the course of upper respiratory tract diseases. Detailed studies require clinical trials with complete identification of cytokine profiles in upper airway secretions and in blood. Furthermore, histological and cytological studies can provide important contributions to understanding the mechanisms of activation of both netosis and etosis. Given the high diversity of patients, to standardize and simplify the study, it seems a good approximation to use a mouse model subjected to inhaled infection with selected pathogens. The obtained results can be used to develop rapid tests to distinguish the basis of airway obstruction, based on the level of NETs/EETs markers. Such identification will allow the implementation of targeted therapy to reduce the proportion of eDNA in the secretion through DNAase degradation or inhibition of netosis/etosis activation mechanisms.In conclusion, NETs appear to play a significant regulatory role in the physicochemical properties of upper respiratory tract secretions. On the one hand, this affects patients’ quality of life and course of illness, while on the other hand, it can be an effective barrier against microorganisms. Overproduction of secretions in an allergic reaction seems to be more beneficial to removing and flushing the mucosa with liquid secretions, hence the eDNA and protein content of NETs is low. Further research into the pharmacological control of NETs in the upper respiratory tract may provide new therapeutic approaches to improve patient’s quality of life.

## Data availability statement

The datasets presented in this study can be found in online repositories. The names of the repository/repositories and accession number(s) can be found below: https://doi.org/10.57903/UJ/PXAMYI.

## Ethics statement

Ethical approval was not required for the studies involving humans because According to the ethics committee’s guidelines, research approval is not required when samples are used as “medical waste” that was previously used for routine diagnostic testing. The research presented in this article was conducted on such a type of sample. The studies were conducted in accordance with the local legislation and institutional requirements. The human samples used in this study were acquired from a by- product of routine care or industry. Written informed consent to participate in this study was not required from the participants or the participants’ legal guardians/next of kin in accordance with the national legislation and the institutional requirements.

## Author contributions

MZ: Conceptualization, Data curation, Formal Analysis, Funding acquisition, Investigation, Methodology, Project administration, Resources, Validation, Visualization, Writing – original draft, Writing – review & editing. MJ: Data curation, Investigation, Methodology, Writing – review & editing. MR-K: Conceptualization, Supervision, Writing – review & editing. JM-W: Validation, Writing – review & editing.
